# Supplementary material for: Cyclin E1 in Murine and Human Liver Cancer: A Promising Target for Therapeutic Intervention during Tumour Progression
Source: Cancers (Basel). 2021 Nov 13;13(22):5680. doi: 10.3390/cancers13225680 (PMC8616292; doi:10.3390/cancers13225680)
Supplement: Supplementary file 1 [file cancers-13-05680-s001.zip › cancers-1412504-supplementary.pdf]

Supplementary Material: Cyclin E1 in Murine and Human Liver Cancer: A Promising Target for Therapeutic Intervention during Tumour Progression

Roland Sonntag, Christian Penners, Marlene Kohlhepp, Ute Haas, Daniela Lambertz,Andreas Kroh, Thorsten Cramer, Fabio Ticconi, Ivan G. Costa, Frank Tacke, Nikolaus Gassler, Christian Trautwein and Christian Liedtke

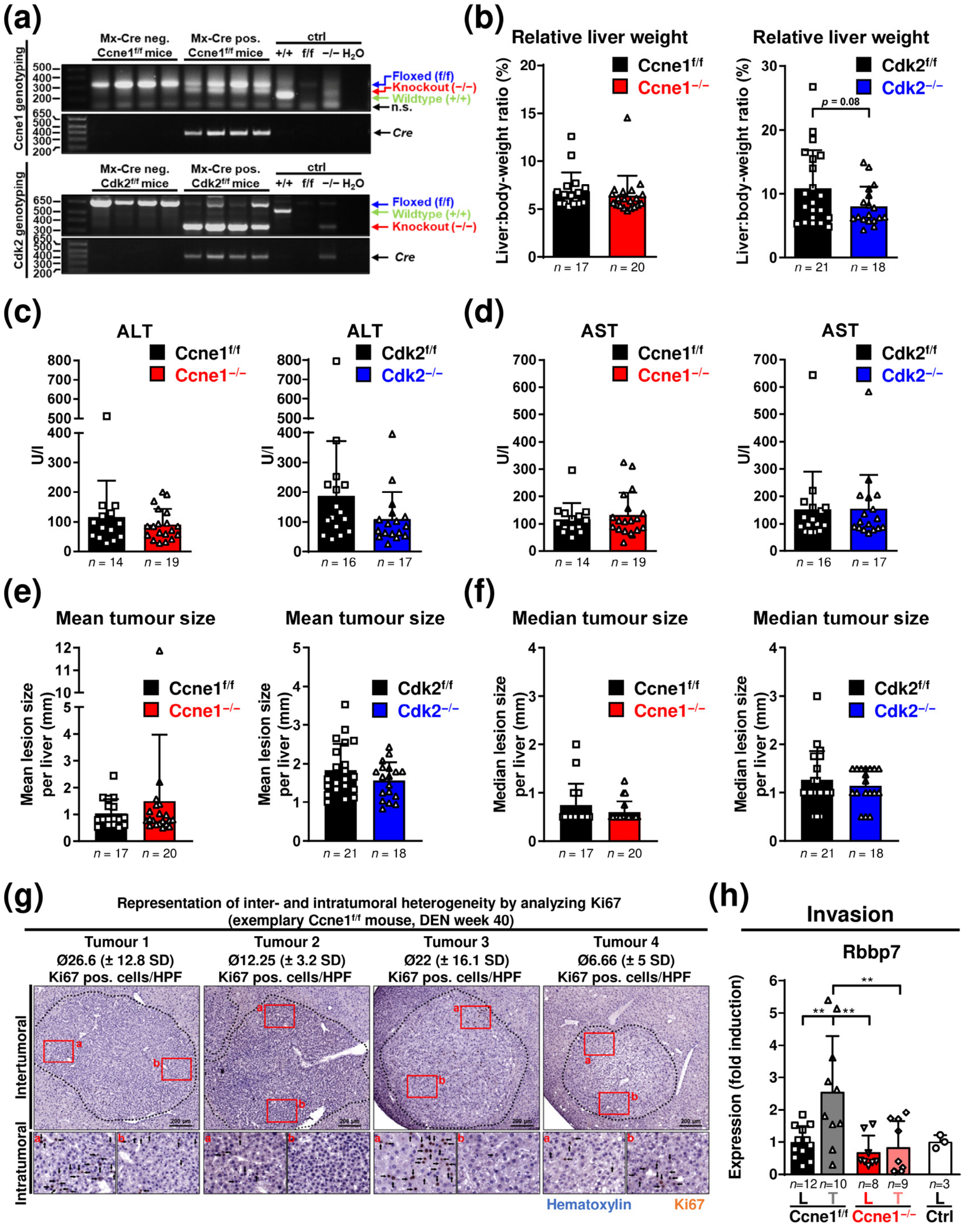

Figure S1. Interventional deletion of *Ccne1*, but not of *Cdk2*, restricts DEN-induced tumour progression *in vivo*. Two-week-old, *Mx-Cre* transgenic mice with floxed *Ccne1* or *Cdk2* alleles were treated with DEN. After tumour initiation, mice were repetitively injected with pIpC at week 22 to induce the deletion of *Ccne1* or *Cdk2* (Ccne1$^{-/-}$, Cdk2$^{-/-}$). *Mx-Cre* negative mice

also received both DEN and pIpC and served as wildtype controls (Ccne1$^{f/f}$, Cdk2$^{f/f}$). HCC progression was analysed after week 40. Details are given in Figure 1a and the method section. (**a**) Verification of the genomic properties and pIpC-induced deletion efficiency from liver extracts of Ccne1$^{f/f}$, Ccne1$^{-/-}$, Cdk2$^{f/f}$ and Cdk2$^{-/-}$ animals 38 weeks after DEN. Top: Genotyping for floxed (f/f, blue), deleted (−/−, red) or wildtype (+/+, green) variants of the *Ccne1* alleles and the presence of the *Cre*-recombinase transgene (black arrow). Bottom: Genotyping for floxed (f/f, blue), deleted (−/−, red) or wildtype (+/+, green) variants of the *Cdk2* alleles and the presence of the *Cre*-recombinase transgene (black arrow). +/+ ctrl: *Cre*-negative, homozygous wildtype control, f/f ctrl: *Cre*-negative Ccne1$^{f/f}$ or Cdk2$^{f/f}$ control, −/− ctrl: Constitutive Ccne1$^{-/-}$ / *Cre*-positive Cdk2$^{-/-}$ controls, H_2O: Water control. (**b**) Liver mass index calculated as the liver:body weight ratio (%) for each Ccne1$^{f/f}$ (left), Ccne1$^{-/-}$ (left) or Cdk2$^{f/f}$ and Cdk2$^{-/-}$ (right) animal. (**c–d**) Determination of (**c**) alanine aminotransferase (ALT) and (**d**) aspartate aminotransferase (AST) activities in mouse serum. (**e**) Mean liver tumour diameter (size, mm) and (**f**) the median liver tumour diameter (size, mm) for each Ccne1$^{f/f}$ and Ccne1$^{-/-}$ or Cdk2$^{f/f}$ and Cdk2$^{-/-}$ animal. (**g**) Immunohistochemical staining for Ki67 (brown) in paraffin sections of one exemplary Ccne1$^{f/f}$ animal demonstrating the heterogeneity of tumour growth. Total nuclei were stained with hematoxylin (blue). The intertumoral variation between four individual tumours (Tumour 1–4) is represented by mean values (Ø) of Ki67-positive cells at a magnification of 200x. The intratumoral variation for each tumour is represented by the standard deviation (± SD) of Ki67-positive cells. Characteristic tissue areas are enlarged (a, b). (**h**) Gene expression analyses in Ccne1$^{f/f}$ and Ccne1$^{-/-}$ animals for *Rbbp7*. L: Liver; T: Tumour tissue, Ctrl: Livers of untreated wildtype mice. Normalized expression levels were calculated as fold induction in comparison to DEN-treated control livers. Corresponding signalling pathways of the target genes are indicated in the headings. Sample sizes are indicated within diagrams. Data are expressed as mean ± SD. **: $p < 0.005$.

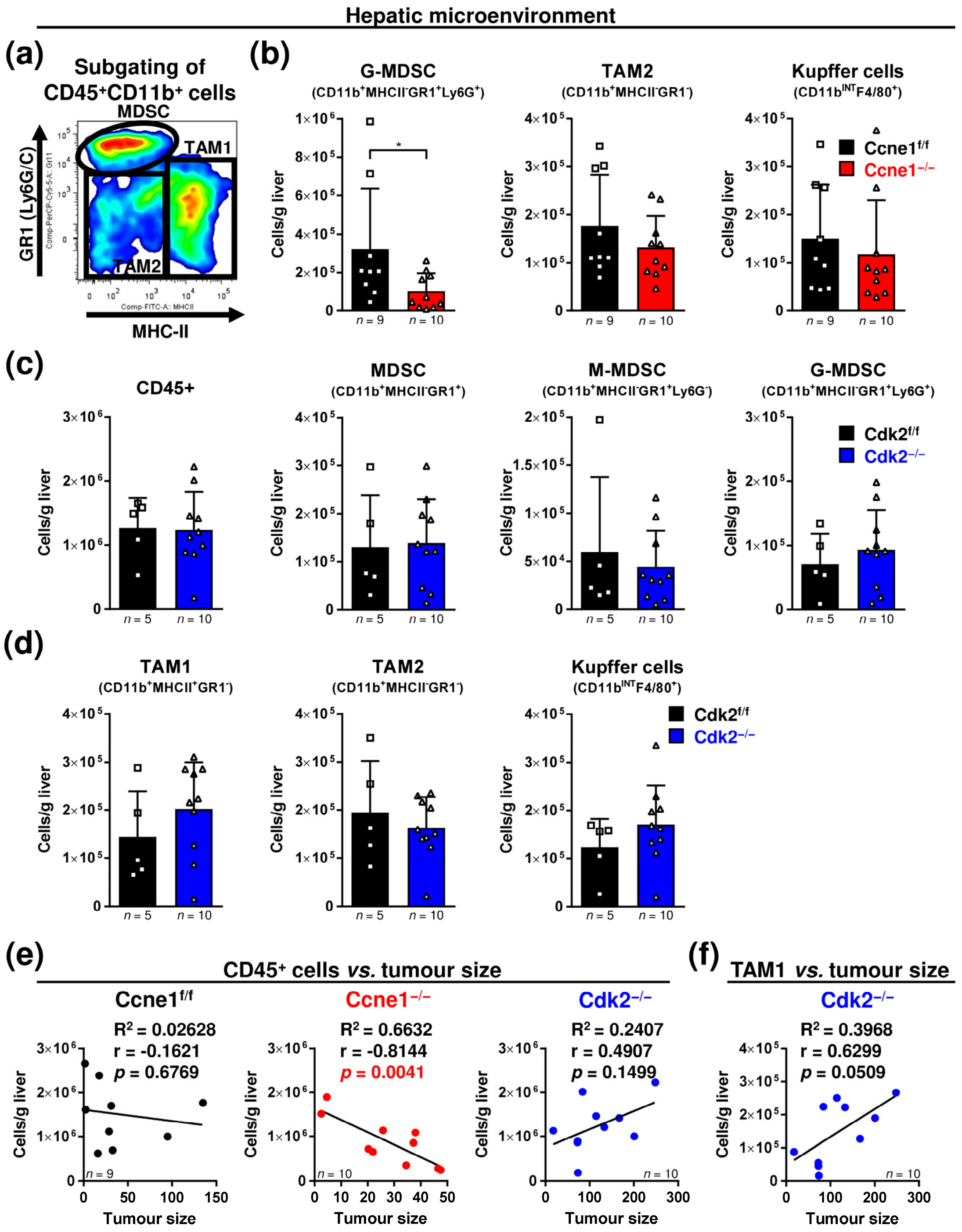

Figure S2. Analysis of the myeloid immune cell compartment in the HCC microenvironment of DEN-treated *Ccne1* or *Cdk2*-deficient mice. *Mx-Cre*-positive mice with floxed *Ccne1* or *Cdk2* alleles and *Cre*-negative control mice were treated with DEN at the age of 2 weeks to induce HCC. At the age of 24 weeks, mice were 3 times injected with pIpC to delete *Ccne1* or *Cdk2* in *Mx-Cre*-positive mice (Ccne1$^{-/-}$, Cdk2$^{-/-}$). PIpC treated *Mx-Cre*-negative mice served as wildtype controls (Ccne1$^{f/f}$, Cdk2$^{f/f}$). HCC progression was analysed at week 40. Details are given in Figure 1a and the method section. (**a**–**d**) Determination of immune cell populations by flow cytometry analysis. (**a**) Gating strategy for CD45/CD11b-positive MDSC, TAM1, TAM2 according to the expression of GR1 (Ly6G/C, PerCP-Cy5.5-A) and MHCII (FITC-A) was performed after Kupffer cell exclusion. (**b**) Number of G-MDSCs, TAM2 and Kupffer cells per gram liver of Ccne1$^{f/f}$ and Ccne1$^{-/-}$ mice. (**c**) Number of CD45-positive cells, MDSCs, M-MDSCs, G-MDSCs, (**d**) TAM1, TAM2 and Kupffer cells and per gram liver of Cdk2$^{f/f}$ and Cdk2$^{-/-}$ mice. Data are expressed as mean ± SD. *: $p < 0.05$. (**e**) Linear analysis of the correlation between tumour size and number of CD45-positive cells per gram liver in Ccne1$^{f/f}$ (black), Ccne1$^{-/-}$ (red) or Cdk2$^{-/-}$ (blue) mice. (**f**) Linear analysis of the correlation between tumour size and number of TAM1-positive cells per gram liver in Cdk2$^{-/-}$ (blue) mice. R^2: Coefficient of determination, r: Pearson correlation coefficient, *p* - values are indicated within diagrams. (**g**) Gene expression analysis of *Ccl2* and *Ccl5* in Ccne1$^{f/f}$ or Ccne1$^{-/-}$ mice. L: Liver; T: Tumour tissue; Ctrl:

Livers of untreated wildtype mice. Normalized expression levels were calculated as fold induction in comparison to DEN-treated control livers. Sample sizes are indicated within diagrams. Data are expressed as mean ± SD. *: $p < 0.05$.

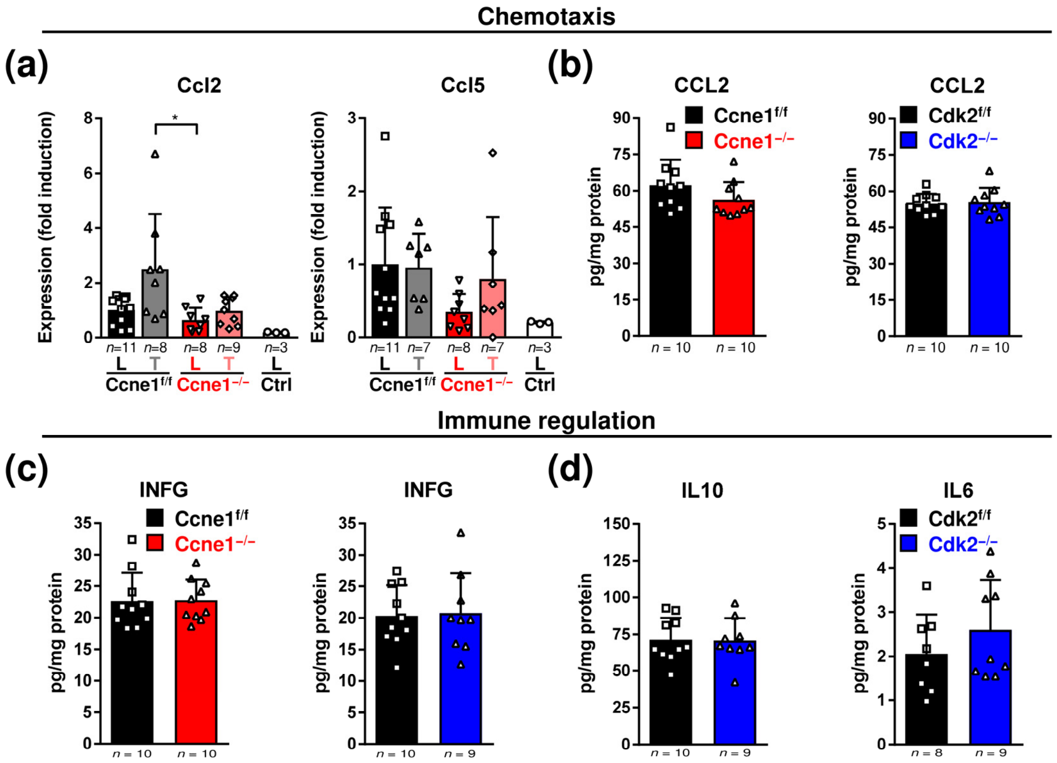

Figure S3. Determination of immune mediators for recruitment, activation and response of myeloid immune cells in the HCC microenvironment of DEN-treated *Ccne1* or *Cdk2*-deficient mice. *Mx-Cre*-positive mice with floxed *Ccne1* or *Cdk2* alleles and *Cre*-negative control mice were treated with DEN at the age of 2 weeks to induce HCC. At the age of 24 weeks, mice were 3 times injected with pIpC to delete *Ccne1* or *Cdk2* in *Mx-Cre*-positive mice ($Ccne1^{-/-}$, $Cdk2^{-/-}$). PIpC treated *Mx-Cre*-negative mice served as wildtype controls ($Ccne1^{f/f}$, $Cdk2^{f/f}$). HCC progression was analysed at week 40. Details are given in Figure 1a and the method section. (**a**) Gene expression analysis of *Ccl2* and *Ccl5* in $Ccne1^{f/f}$ or $Ccne1^{-/-}$ mice. L: Liver; T: Tumour tissue; Ctrl: Livers of untreated wildtype mice. Normalized expression levels were calculated as fold induction in comparison to DEN-treated control livers. (**b–c**) Determination of (b**)** CCL2, (**c**) INFG in livers of $CcnE1^{f/f}$ and $CcnE1^{-/-}$ (left) or $Cdk2^{f/f}$ and $Cdk2^{-/-}$ (right) animals by ELISA. (**d**) Determination of IL10 and IL6 concentrations in DEN-treated livers of $Cdk2^{f/f}$ and $Cdk2^{-/-}$ animals by ELISA. Corresponding signalling pathways of the target genes are indicated in the headings. Data are expressed as mean ± SD. Sample sizes are indicated within diagrams. *: $p < 0.05$.

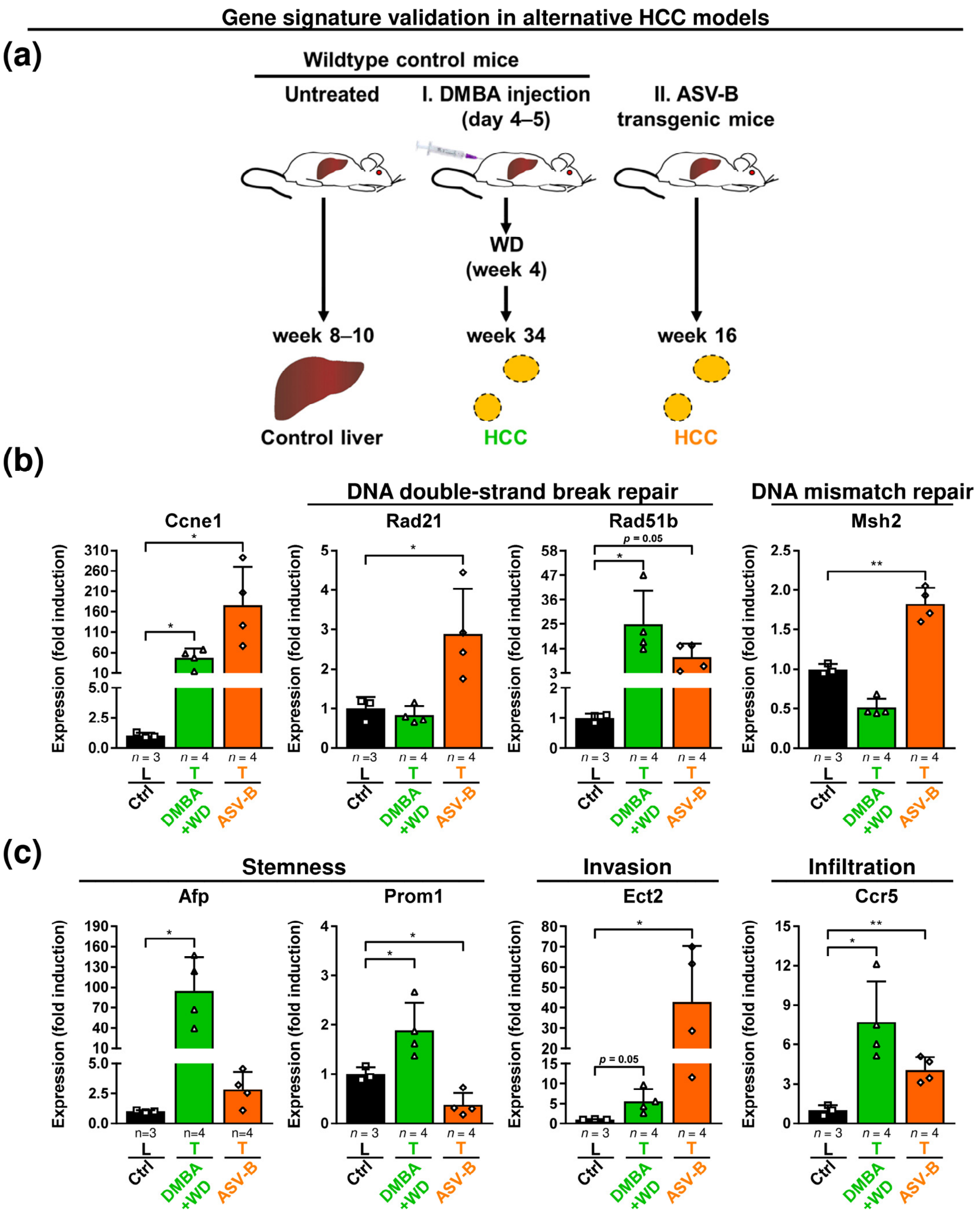

Figure S4. Murine HCC progression shares a common signature of enhanced *Ccne1* expression and markers of DNA damage repair, stemness and leucocyte infiltration. The verification of a common gene expression signature, which was obtained from the DEN-induced model of hepatocarcinogenesis, was carried out in tumour samples from two additional, independent HCC mouse models. (**a**) Schematic representation of the experimental approach. In the first investigated model (I), HCC development was driven by the carcinogen 7,12-Dimethylbenz[a]anthracene (DMBA, injected at postnatal day 4–5) combined with a Western diet (WD)-induced non-alcoholic steatohepatitis (NASH, 30 weeks of feeding) in C57B6/J-wildtype mice (Ctrl). Dissected HCCs were analysed in comparison to untreated livers of 8 to 10-week-old C57B6/J-wildtype mice. The second HCC model (II) comprised ASV-B transgenic mice expressing the simian virus 40 large and small T antigens under the control of the antithrombin III regulatory sequences. At the age of 16 weeks, mice were sacrificed and dissected HCCs were analysed in comparison to untreated livers of 8 to 10-week-old C57B6/J-wildtype mice (Ctrl). (**b–c**) Gene expression analysis by qPCR. (**b**) Analysis of *Ccne1*, *Rad51b*, *Rad21*, *Msh2*. (**c**) Analysis of *Afp*, *Prom1*, *Ect2* and *Ccr5*. Corresponding signalling pathways of the target genes are indicated in the headings. Normalized expression levels were calculated as fold induction in comparison to untreated control livers. Sample sizes are indicated within diagrams. Data are expressed as mean ± SD. *: $p < 0.05$; **: $p < 0.005$.

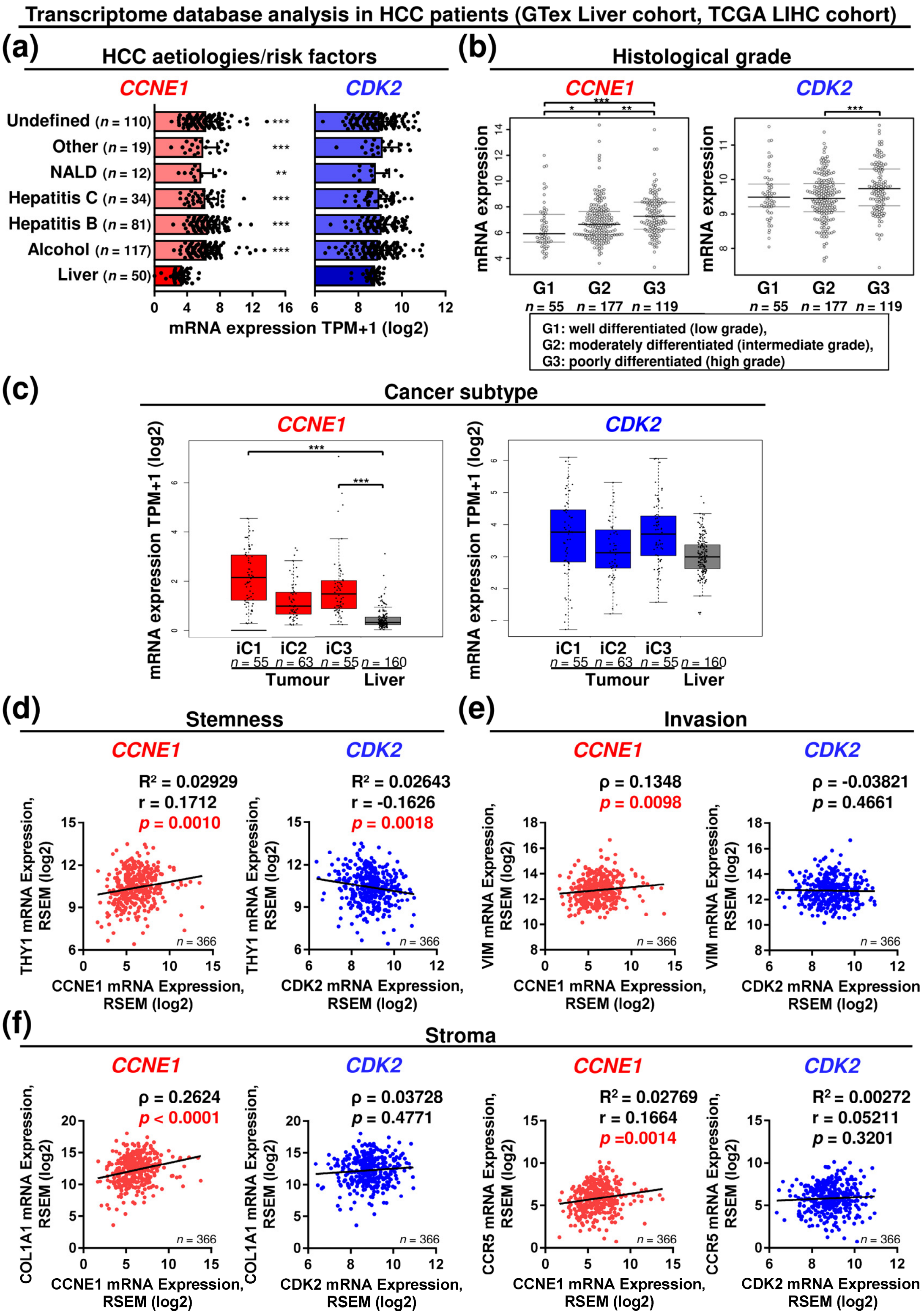

Figure S5. *CCNE1* expression is a diagnostic and prognostic parameter in human HCC subtypes sharing high levels of dedifferentiation, invasion, immune cell recruitment and chromosomal instability. *In silico* database analysis of *CCNE1* and *CDK2* expression in tumour samples and matching tumour free-liver samples of HCC patients based on RNA-seq data generated by the TCGA Research Network, LIHC cohort (http://cancergenome.nih.gov/, *n* = 373 HCC samples, *n* = 50 liver samples) and independent tumour-free liver samples (*n* = 110) by GTex liver cohort (http://gtexportal.org/). Details are given in the method section. (**a**) mRNA expression levels of *CCNE1* (left) and *CDK2* (right) in patient healthy livers and HCC according to aetiology history. TPM: Transcript per kilobase million. (**b**) The mRNA expression levels of *CCNE1* (left) and *CDK2* (right) in HCC samples of different histological grades (G1, G2, G3). (**c**) *CCNE1* (left) and *CDK2* (right) mRNA expression in tumour-free liver samples and HCC subtypes according to iCluster grouping (iC1, iC2, iC3, according to http://gepia2.cancer-pku.cn/). Data are expressed as mean ± SD. *: $p < 0.05$; **: $p < 0.005$; ***: $p < 0.001$. (**d–f**) Linear

analysis in human HCC patients. (**d**) Correlation analysis between mRNA expression levels of *CCNE1* (left) or *CDK2* (right) and *THY1* mRNA expression in HCC patients. (**e**) Correlation analysis between mRNA expression levels of *CCNE1* (left) or *CDK2* (right) and *VIM* mRNA expression in HCC patients. (**f**) Left: Correlation analysis between mRNA expression levels of *CCNE1* or *CDK2* and *COL1A1* mRNA expression in HCC patients. Right: Correlation between mRNA expression levels of *CCNE1* or *CDK2* and *CCR5* mRNA expression. Corresponding signalling pathways of the target genes are indicated in the headings. R^2: Coefficient of determination, r: Pearson correlation coefficient, ϱ: Spearman correlation coefficient. *P* - values and sample sizes are indicated within diagrams. RSEM: RNA-Seq by Expectation-Maximization.

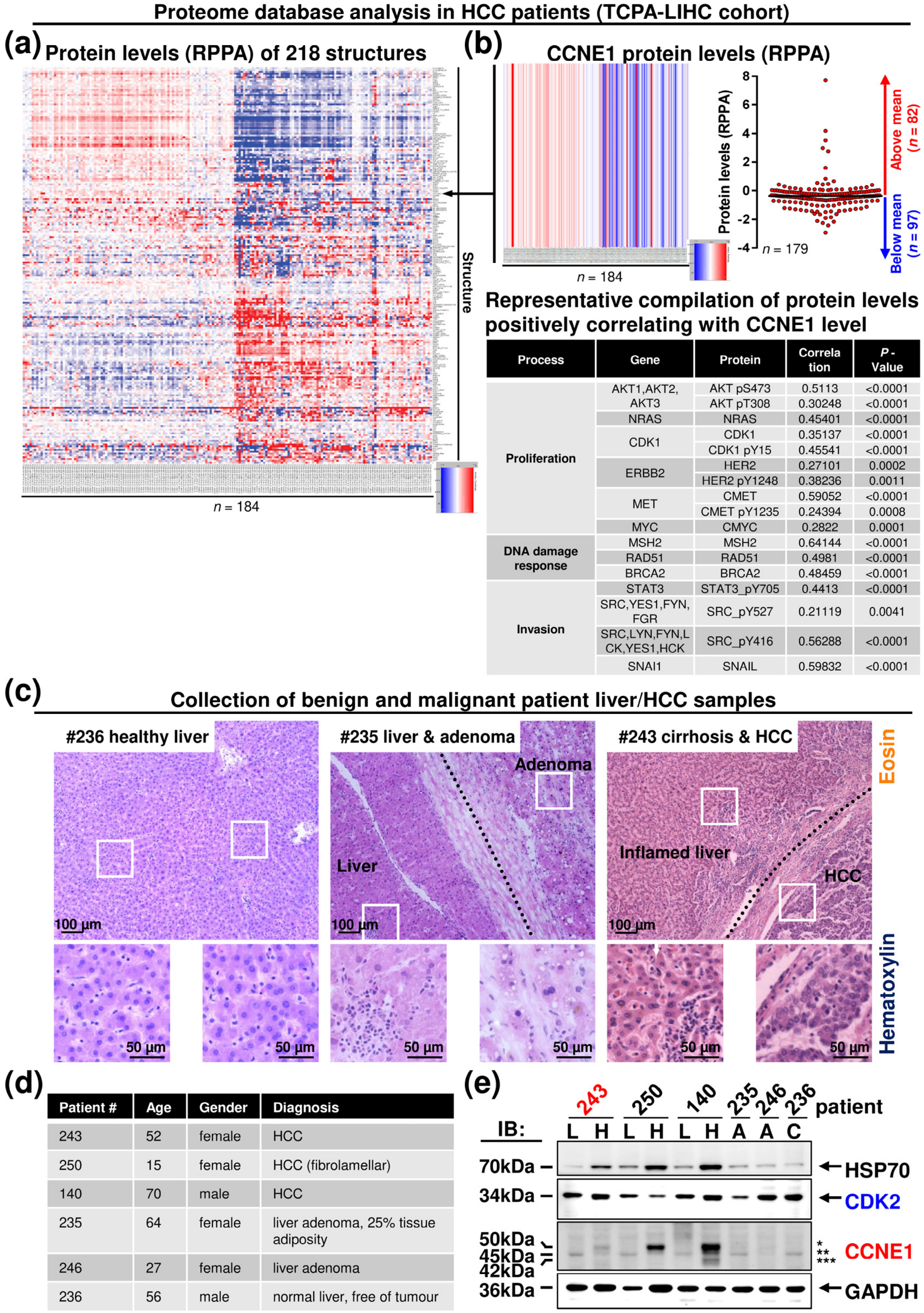

Process	Gene	Protein	Correlation	P-Value
Proliferation	AKT1,AKT2, AKT3	AKT pS473	0.5113	<0.0001
		AKT pT308	0.30248	<0.0001
	NRAS	NRAS	0.45401	<0.0001
	CDK1	CDK1	0.35137	<0.0001
		CDK1 pY15	0.45541	<0.0001
	ERBB2	HER2	0.27101	0.0002
		HER2 pY1248	0.38236	0.0011
	MET	CMET	0.59052	<0.0001
		CMET pY1235	0.24394	0.0008
	MYC	CMYC	0.2822	0.0001
DNA damage response	MSH2	MSH2	0.64144	<0.0001
	RAD51	RAD51	0.4981	<0.0001
	BRCA2	BRCA2	0.48459	<0.0001
Invasion	STAT3	STAT3_pY705	0.4413	<0.0001
	SRC,YES1,FYN, FGR	SRC_pY527	0.21119	0.0041
	SRC,LYN,FYN,LCK,YES1,HCK	SRC_pY416	0.56288	<0.0001
	SNAI1	SNAIL	0.59832	<0.0001

Patient #	Age	Gender	Diagnosis
243	52	female	HCC
250	15	female	HCC (fibrolamellar)
140	70	male	HCC
235	64	female	liver adenoma, 25% tissue adiposity
246	27	female	liver adenoma
236	56	male	normal liver, free of tumour

Figure S6. CCNE1 protein levels in advanced human HCCs are associated with markers of proliferation, DNA damage response and invasion. (**a**–**b**) *In silico* proteome database analysis of 218 target protein structures in individual HCC

patients of the LIHC cohort (n = 184) based on reverse phase protein array (RPPA) generated by the The Cancer Protein Atlas (TCPA) portal. Details are given in the method section. (**a**) Heatmap representation of the total protein RPPA levels in the cohort (available at https://www.tcpaportal.org/). The arrow highlights CCNE1 (CYCLINE1). (**b**) Top: Heatmap (left) and scatter plot (right, according to http://cbioportal.org/) representation of the CCNE1 protein RPPA levels in the cohort. Red arrow and bars: Patients with CCNE1 levels above mean value; blue arrow and bars: Patients with CCNE1 levels below mean value. Bottom: Table of representative total and phosphorylated protein structures positively correlating with CCNE1 after analysis of RPPA levels in HCC samples. P - values and correlation coefficient are given within table (complete data are available at https://www.tcpaportal.org/). (**c–e**) Analysis of a small cohort of human hepatocellular carcinoma samples (H) and matching surrounding liver (L) tissues (#243, 250, 140), adenomas (A, #235, 246) and healthy control liver (C, #236). (**c**) H&E-stained liver sections of selected patients displaying healthy control liver (#236), benign liver adenoma (#235) or HCC (#243). Dashed lines highlight the border between tumorous and non-tumorous tissue. Characteristic tissue areas are enlarged. (**d**) Table of patient details. (**e**) Immunoblot analysis of CCNE1, CDK2 and the HCC biomarker heat-shock protein 70 (HSP70) from whole liver extracts of non-tumorous liver tissue (L), matching HCC (H), benign liver adenoma (A) or healthy control tissue (C). Asterisks (*, **, ***) indicate different CCNE1 isoforms (~50 kDa, ~45kDa, ~42 kDa). GAPDH served as internal loading control.

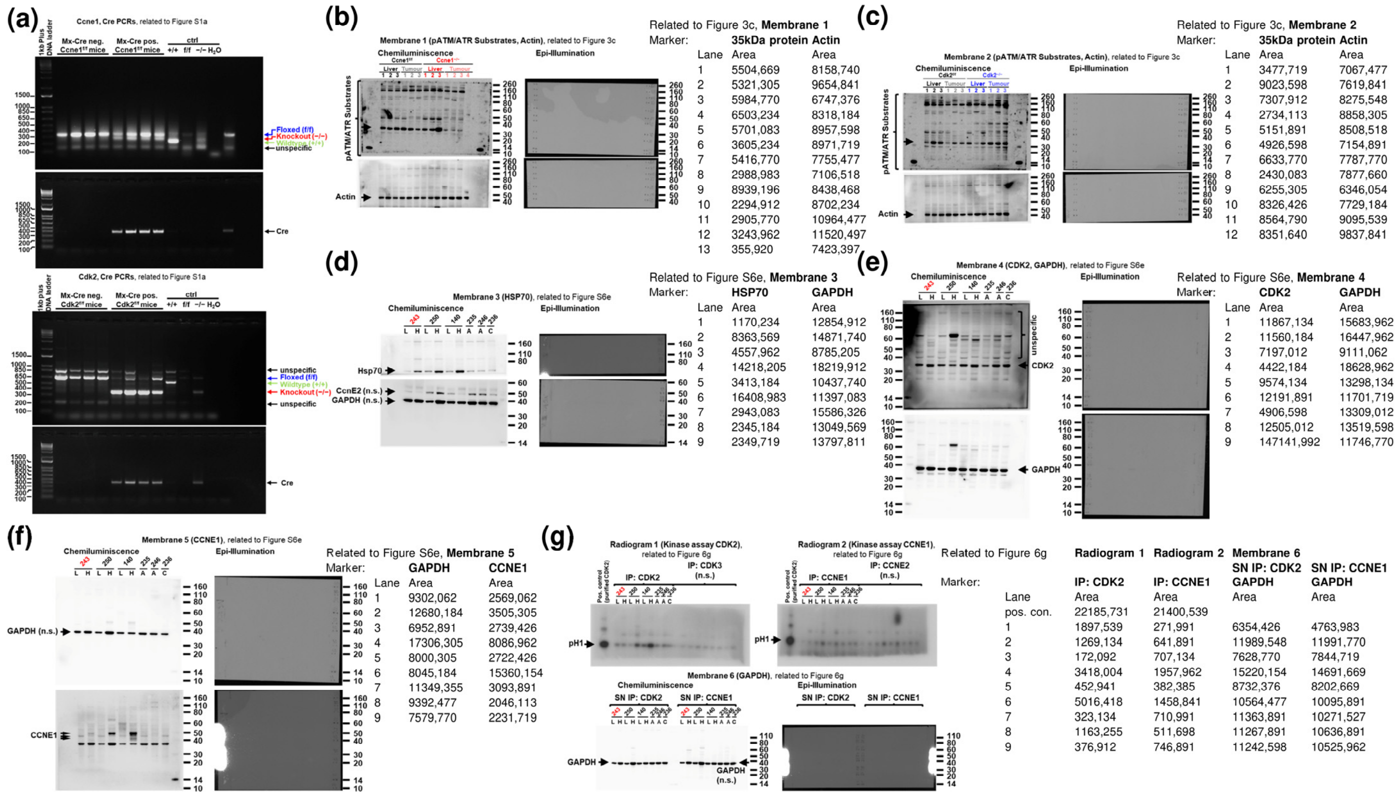

Related to Figure 3c, **Membrane 1**

Marker:	**35kDa protein**	**Actin**
Lane	Area	Area
1	5504,669	8158,740
2	5321,305	9654,841
3	5984,770	6747,376
4	6503,234	8318,184
5	5701,083	8957,598
6	3605,234	8971,719
7	5416,770	7755,477
8	2988,983	7106,518
9	8939,196	8438,468
10	2294,912	8702,234
11	2905,770	10964,477
12	3243,962	11520,497
13	355,920	7423,397

Related to Figure 3c, **Membrane 2**

Marker:	**35kDa protein**	**Actin**
Lane	Area	Area
1	3477,719	7067,477
2	9023,598	7619,841
3	7307,912	8275,548
4	2734,113	8858,305
5	5151,891	8508,518
6	4926,598	7154,891
7	6633,770	7787,770
8	2430,083	7877,660
9	6255,305	6346,054
10	8326,426	7729,184
11	8564,790	9095,539
12	8351,640	9837,841

Related to Figure S6e, **Membrane 3**

Marker:	**HSP70**	**GAPDH**
Lane	Area	Area
1	1170,234	12854,912
2	8363,569	14871,740
3	4557,962	8785,205
4	14218,205	18219,912
5	3413,184	10437,740
6	16408,983	11397,083
7	2943,083	15586,326
8	2345,184	13049,569
9	2349,719	13797,811

Related to Figure S6e, **Membrane 4**

Marker:	**CDK2**	**GAPDH**
Lane	Area	Area
1	11867,134	15683,962
2	11560,184	16447,962
3	7197,012	9111,062
4	4422,184	18628,962
5	9574,134	13298,134
6	12191,891	11701,719
7	4906,598	13309,012
8	12505,012	13519,598
9	147141,992	11746,770

Related to Figure S6e, **Membrane 5**

Marker:	**GAPDH**	**CCNE1**
Lane	Area	Area
1	9302,062	2569,062
2	12680,184	3505,305
3	6952,891	2739,426
4	17306,305	8086,962
5	8000,305	2722,426
6	8045,184	15360,154
7	11349,355	3093,891
8	9392,477	2046,113
9	7579,770	2231,719

Related to Figure 6g	**Radiogram 1**	**Radiogram 2**	**Membrane 6**	
Marker:	**IP: CDK2**	**IP: CCNE1**	**SN IP: CDK2 GAPDH**	**SN IP: CCNE1 GAPDH**
Lane	Area	Area	Area	Area
pos. con.	22185,731	21400,539		
1	1897,539	271,991	6354,426	4763,983
2	1269,134	641,891	11989,548	11991,770
3	172,092	707,134	7628,770	7844,719
4	3418,004	1957,962	15220,154	14691,669
5	452,941	382,385	8732,376	8202,669
6	5016,418	1458,841	10564,477	10095,891
7	323,134	710,991	11363,891	10271,527
8	1163,255	511,698	11267,891	10636,891
9	376,912	746,891	11242,598	10525,962

Figure S7. Uncropped gels, western blots and kinase assays. (**a**) Uncropped images after agarose gel electrophoresis for PCR-based verification of the genomic properties and pIpC-induced deletion efficiency from liver extracts of Ccne1$^{f/f}$, Ccne1$^{-/-}$, Cdk2$^{f/f}$ and Cdk2$^{-/-}$ animals 38 weeks after DEN, related to Figure S1a. Standard DNA sizes (100–1500 bp) are indicated. (**b–g**) Left: Uncropped membranes and radiograms. Images show chemiluminescence signals obtained after incubation with the corresponding primary and HRP-linked secondary antibody (chemiluminescence), epi-illumination of each membrane or phosphorylation of the substrate H1 (pH1) after kinase reaction, polyacrylamide gel electrophoresis and autoradiography (radiogram). Standard molecular weights (10–260 kDa) are indicated at each membrane. Arrows indicate markers. Right: Tables of densitometric analysis of the corresponding markers according to each lane using ImageJ Gel analyser v.1.53c (http://imagej.nih.gov/ij/, National institute of health, USA). (**b–c**) Western blots related to Figure 3b. (**d–f**) Western blots related to Figure S6e. (**g**) Radiogram and western blots related to Figure 6g. N.s.: Not shown in the corresponding Figure.gels, western blots and kinase assays. **(a)** Uncropped images after agarose gel electrophoresis for PCR-based verification of the genomic properties and pIpC-induced deletion efficiency from liver extracts of Ccne1$^{f/f}$, Ccne1$^{-/-}$, Cdk2$^{f/f}$ and Cdk2$^{-/-}$ animals 38 weeks after DEN, related to Figure S1a. Standard DNAsizes (100-1500 bp) are indicated. **(b-g)** Left: Uncropped membranes and radiograms.Images show chemiluminescence signals obtained after incubation with thecorresponding primary and HRP-linked secondary antibody (chemiluminescence), epi-illumination of each membrane or phosphorylation of the substrate H1 (pH1) after kinase reaction, polyacrylamide gel electrophoresis and autoradiography (radiogram).Standard molecular weights (10-260 kDa) are indicated at each membrane. Arrows indicate markers. Right: Tables of densitometric analysis of the corresponding markersaccording to each lane using ImageJ Gel

analyser v.1.53c (http://imagej.nih.gov/ij, National institute of health, USA). **(b-c)** Western blots related to Figure 3b. **(d-f)** Western blots related to Figure S6e. **(g)** Radiogram and western blots related to Figure6g. N.s.: Not shown in the corresponding Figure.

Table S1. Summary of pathological investigation. *Mx-Cre*-positive mice with floxed*Ccne1* allele and *Cre*-negative control mice were treated with DEN at the age of 2 weeks to induce HCC. At the age of 24 weeks, mice were three times injected with pIpC to delete *Ccne1* in *Mx-Cre*-positive (*Cre* pos.) mice. PIpC treated *Mx-Cre*-negative (*Cre* neg.) mice served as wildtype controls. Mice were analysed at week 40 (N.G.); m: several tumours enumerated for 10 HPF; n.d.: not definable, excluded sample.

Mouse, Cre status	Number of mitoticbodies/10 HPF in tumour tissue	Number of mitoticbodies/10 HPF in non-tumorous tissue	Venous invasion(V1); 0: not detected, 1:detected
**#1, *Cre* neg.**	1	0	1
**#2, *Cre* neg.**	7	0	1
**#3, *Cre* neg**	2	0	0
**#4, *Cre* neg.**	10	1	1
**#5, *Cre* neg.**	11	0	1
**#6, *Cre* neg.**	1	0	1
**#7, *Cre* neg.**	n.d.	0	0
**#8, *Cre* neg.**	2	0	0
**#9, *Cre* neg.**	5	0	1
**#10, *Cre* neg.**	1 (m)	0	0
**#11, *Cre* pos.**	0 (m)	0	0
**#12, *Cre* pos.**	1 (m)	1	0
**#13, *Cre* pos.**	0 (m)	0	0
**#14, *Cre* pos.**	0 (m)	0	0
**#15, *Cre* pos.**	2	0	0
**#16, *Cre* pos.**	0	0	0
**#17, *Cre* pos.**	1	0	0
**#18, *Cre* pos.**	0	0	0
**#19, *Cre* pos.**	2	0	0

Table S2. Summary of iCluster molecular and clinical correlates according toWheeler D.A. and Lewis R. R (2017).

Clinical/MolecularVariable	Categories	iCluster 1	iCluster 2	iCluster 3	*p* value
**Age**	Mean (SD)	54.954 (15.859)	61.164 (14.727)	64.587 (10.067)	0,0005
**Gender**	Female (n(%))	28(43%)	14(25%)	18(29%)	0,09
	Male (n(%))	37(57%)	41(75%)	45(71%)	
**Race**	American Indian	1(2%)	1(2%)	0(0%)	0,002
	Asian	30(47%)	7(14%)	15(24%)	
	African American	3(5%)	4(8%)	7(11%)	
	White	30(47%)	37(76%)	40(65%)	
**Weight**	Normal	42(76%)	20(42%)	18(34%)	5,00E-05
	Obese	8(15%)	13(27%)	13(25%)	
	Overweight	5(9%)	15(31%)	22(42%)	
**Grade**	G1	5(8%)	17(32%)	10(16%)	0,0006
	G2	28(43%)	27(51%)	32(52%)	
	G3	32(49%)	9(17%)	20(32%)	
**MacrovascularInvasion**	Yes	5(10%)	0(0%)	3(6%)	0,01
	MicrovascularInvasion	18(35%)	7(15%)	18(33%)	
	None	29(56%)	39(85%)	33(61%)	
***CTNNB1* mutation**		8(12%)	21(38%)	27(43%)	0,0002
***TERT* promotermutation**		14(22%)	31(56%)	34(55%)	7,24E-05
***TP53* mutation**		16(25%)	13(25%)	28(45%)	0,03

**Hoshida**	1	15(23%)	11(20%)	1(2%)	
	2	46(71%)	17(31%)	20(32%)	5,59E-14
	3	4(6%)	27(49%)	42(67%)	
**Age > 65**	No	48(74%)	30(55%)	31(49%)	0,01
	Yes	17(26%)	25(45%)	32(51%)	

Table S3. Primer sequences for quantitative real-time PCR.

Gene	Origin	Orientation	Sequence in 5´-3´orientation
Afp	murine	sense	AGCAAAGCTGCGCTCTCTAC
		antisense	AGGGGCTTTCCTCGTGTAAC
Ccl2	murine	sense	GTGTTGGCTCAGCCAGATGC
		antisense	GACACCTGCTGCTGGTGATCC
Ccl5	murine	sense	GCTGCTTTGCCTACCTCTCC
		antisense	TCGAGTGACAAACACGACTGC
Ccna2	murine	sense	GTGGTGATTCAAAACTGCCA
		antisense	AGAGTGTGAAGATGCCCTGG
Ccnd1	murine	sense	AAGCATGCACAGACCTTTGTGG
		antisense	TTCAGGCCTTGCATCGCAGC
Ccne1	murine	sense	TCCACGCATGCTGAATTATC
		antisense	TTGCAAGACCCAGATGAAGA
Ccne2	murine	sense	AAAAAGTCTTGGGCAAGGTAAA
		antisense	GCATTCTGACCTGGAACCAC
Ccr2	murine	sense	TCGCTGTAGGAATGAGAAGAAGAGG
		antisense	CAAGGATTCCTGGAAGGTGGTCAA
Ccr5	murine	sense	GTCAGAACGGTCAACTTTGGG
		antisense	GTGTGGAAAATGAGGACTGCAT
Cd274	murine	sense	CCTCGCCTGCAGATAGTTC
		antisense	CCTTTGGAGCCGTGATAG
Col1a1	murine	sense	TCTGACTGGAAGAGCGGAGAG
		antisense	GGCACAGACGGCTGAGTAGG
Ect2	murine	sense	GGATTCTGGATCAGCCACAT
		antisense	ACGTCAGAGGAGCTTCCAAA
Rad51b	murine	sense	CAATCCCAGTTACATTTCAACCGAT
		antisense	ACGACTTCCCACCTCAGTG
Gapdh	murine	sense	TGTTGAAGTCACAGGAGACAACCT
		antisense	AACCTGCCAAGTATGATGACATCA
Icam1	murine	sense	GCCAGGAGCCGGACTTTCG
		antisense	GGCAGGAAACAGGCCTTCCAGGG
Il10	murine	sense	GCCAAGCCTTATCGGAAATGA
		antisense	CTTGATTTCTGGGCCATGCT
Msh2	murine	sense	TCCATCCTCAGGTCAGCAAC
		antisense	TGGGTGGCAAACATGCAAAA
Prom1	murine	sense	CAAACCCATGGCCACCGCGA
		antisense	CACCGTGGCTTTCCCTATGCCG
Rad21	murine	sense	GCCGAGATCCAGGTTTCTTC
		antisense	ACATGGGCTTTGGTTAGCTTC
Rbbp7	murine	sense	TTCAAACATCTCTTTACTCGCCATC
		antisense	TTCAAACATCTCTTTACTCGCCATC
Snai1	murine	sense	CCCTTCAGGCCACCTTCTTT
		antisense	ATGTGTCCAGTAACCACCCTG

Table S4. Primer sequences for genotyping PCR.

Gene	Primer	Sequence in 5´-3´orientation	Combina-tion	Signal (length)
Ccne1	P1	CGCATACTGAGACACAGACT	P1 + P2	WT (230 bp)
	P2	CGCCATGGTTATCCGGGAGATGG	P1 + P3	KO (310 bp)/floxed(350 bp)
	P3	GAAGAGGGCATCAGATCCTATTAC		
Cdk2	P1	CAAGTTGACGGGAGAAGTTGTG	P1 + P2	WT (560 bp)
	P2	GAAGACCCTCCAGGTGAATGAA	P1 + P3	KO (360 bp)/floxed(670 bp)
	P3	GCGATAAGCTTCGAGGGACC		
Cre	P1	CCACGACCAAGTGACAGCAAT	P1 + P2	Cre (392 bp)
	P2	TTCGGATCATCAGCTACACCA		

Table S5. Antibodies for Western Blot, Immunofluorescence and flowcytometry.

Product	Company
**Primary antibodies (non-conjugated)**	
Alpha Smooth Muscle Actin [1A4], A2547	Sigma-Aldrich
HSP70, MAB1663	R&D Systems
Beta Actin, ab8227	Abcam
Cyclin E1 (M-20), sc-481 or (HE-12), sc-247	Santa Cruz Biotechnology
Cyclin E1 07-687	Merck Millipore
GAPDH (MCA4739)	AbD Serotec
CDK2 (D-12), sc-6248	Santa Cruz Biotechnology
Ki-67 [SP6], ab16667	Abcam
Phospho-Histone H2A.X, Ser139 (20E3), #9718	Cell Signaling Technology
Phospho-(Ser/Thr) ATM/ATR Substrate Antibody, #2851	Cell Signaling Technology
**Primary antibodies (fluorescence-labeled) & dyes**	
Hoechst 33342 Solution, 561908	BD Bioscience
CD11b-BV711 (M1/70), 563168 or CD11b-PerCP-Cy5.5(M1/70), 550993	BD Bioscience
CD11b-PE BD (M1/70), 12-0112-82	Invitrogen Thermo FisherScientific
CD11c-AL700 (HL3), 560583	BD Bioscience
CD11c-APC (N418), 117312	Biolegend
CD19-AL700 (eBio1D3), 56-0193-80 or CD19-PE-Cy5(eBio1D3), 15-0193-81	Invitrogen Thermo FisherScientific
CD25-PerCP-Cy5.5 (PC61), 551071	BD Bioscience
CD3-APC (145-2C11), 17-0031-82 or CD3-PE-Cy7(145-2C11), 25-0031-82	Invitrogen Thermo FisherScientific
CD45-AL700 (30-F11), 56-0451-82	Invitrogen Thermo FisherScientific
CD45-APC-Cy7 (30-F11), 557659	BD Bioscience
CD4-APC (GK1.5), 17-0041-83 or CD4-eFluor450 (GK1.5),48-0041-82	Invitrogen Thermo FisherScientific
CD4-FITC (RM4-5), 553046	BD Bioscience
CD4-PE (GK1.5), 12-0041-83	Invitrogen Thermo FisherScientific
CD8-BV711 (53-6.7),100759	Biolegend
CD8-FITC (53-6.7), 11-0081-85	Invitrogen Thermo FisherScientific
CD8-PE (53-6.7), 553033	BD Bioscience
F4/80-PE-Cy7 (BM8), 15-4801-82	Invitrogen Thermo FisherScientific
GR-1-APC (RB6-8C5), 17-5931-82 or GR-1-PE (RB6-8C5),12-5931-83	BD Bioscience
GR-1-PerCP-Cy5.5 (RB6-8C5), 552093	Invitrogen Thermo FisherScientific
Ly6G-AL700 (1A8), 127622 or Ly6G-PE (1A8), 551461	Biolegend
Ly6G-APC (1A8), 17-9668-82	Invitrogen Thermo FisherScientific
MHCII-eFluor450 (M5/114.15.2), 48-5321-82	Invitrogen Thermo FisherScientific
MHCII-FITC (M5/114.15.2), 107605	Biolegend
NK1.1-APC (PK136), 550627	BD Bioscience
NK1.1-APC-Cy7 (PK136), 108724	Biolegend

NK1.1-PE-Cy7 (PK136), 25-5941-82	Invitrogen Thermo FisherScientific
TCRß-PE-Cy7 (H57-597), 25-5961-82	Invitrogen Thermo FisherScientific
7-AAD, 559925	BD Bioscience
**Secondary antibodies (HRP-conjugated)**	
mouse anti-rabbit IgG-HRP, sc-2357	Santa Cruz Biotechnology
m-IgGκ BP-HRP, sc-516102	Santa Cruz Biotechnology
m-IgGλ BP-HRP, sc-516132	Santa Cruz Biotechnology
ImmPRESS HRP Goat Anti-Rabbit IgG Polymer DetectionKit, Peroxidase	Vector Laboratories
**Secondary antibodies (fluorescence-labeled)**	
Alexa Fluor 488 goat anti-rabbit IgG, A-11008	Invitrogen Thermo FisherScientific
Alexa Fluor 594 donkey anti-rabbit IgG, A-21207	Invitrogen Thermo FisherScientific
